# Discovery of an ultraspecific triuret hydrolase (TrtA) establishes the triuret biodegradation pathway

**DOI:** 10.1074/jbc.RA120.015631

**Published:** 2020-12-01

**Authors:** Lambros J. Tassoulas, Mikael H. Elias, Lawrence P. Wackett

**Affiliations:** 1 Department of Biochemistry, Biophysics & Molecular Biology, University of Minnesota, Minneapolis, Minnesota, USA; 2 BioTechnology Institute, University of Minnesota, St Paul, Minnesota, USA

**Keywords:** TrtA, triuret, biuret;, BiuH, substrate discrimination, enzyme evolution, cysteine hydrolase, IHL protein family, nitrogen-containing compound, uric acid oxidation, urea pyrolysis, BiuH, Biuret hydrolase, BME, *beta*-mercaptoethanol, CD, circular dichroism, FPLC, fast protein liquid chromatography, IHL, isochorismatase-like hydrolase, LB, lysogeny broth, VDW, van der Waals

## Abstract

Triuret (carbonyldiurea) is an impurity found in industrial urea fertilizer (<0.1% w/w) that is applied, worldwide, around 300 million pounds each year on agricultural lands. In addition to anthropogenic sources, endogenous triuret has been identified in amoeba and human urine, the latter being diagnostic for hypokalemia. The present study is the first to describe the metabolic breakdown of triuret, which funnels into biuret metabolism. We identified the gene responsible for triuret decomposition (*trtA*) in bacterial genomes, clustered with *biuH*, which encodes biuret hydrolase and has close protein sequence homology. TrtA is a member of the isochorismatase-like hydrolase (IHL) protein family, similarly to BiuH, and has a catalytic efficiency (k_cat/_K_M_) of 6 x 10^5^ M^−1^s^−1^, a K_M_ for triuret of 20 μM, and exquisite substrate specificity. Indeed, TrtA has four orders of magnitude less activity with biuret. Crystal structures of TrtA in apo and holo form were solved and compared with the BiuH structure. The high substrate selectivity was found to be conveyed by second shell residues around each active site. Mutagenesis of residues conserved in TrtA to the alternate consensus found in BiuHs revealed residues critical to triuret hydrolase activity but no single mutant evolved more biuret activity, and likely a combination of mutations is required to interconvert between TrtA, BiuH functions. TrtA-mediated triuret metabolism is relatively rare in recorded genomes (1–2%), but is largely found in plant-associated, nodulating, and endophytic bacteria. This study suggests functions for triuret hydrolase in certain eukaryotic intermediary processes and prokaryotic intermediary or biodegradative metabolism

Triuret (carbonyldiurea) has been described in the literature as early as 1870 when it was first synthesized (1). Although the biosynthesis of triuret remains to be elucidated, it has been observed in some freshwater amoebas in crystalline form where it perhaps serves as a novel nitrogen storage form with low water solubility (∼1 mM, 25 ^o^C) (2). Triuret has also been detected in humans in urine under conditions of high oxidative stress and has a suggested linkage to hypokalemia (2, 3, 4). While the natural sources of triuret are unknown, it is hypothesized to come from oxidative purine or uric acid metabolism (3, 8, 12, 13, 14).

Anthropogenic sources include triuret as an impurity of industrial urea fertilizer (∼0.1% w/w) for agriculture, the result of pyrolysis of urea during fertilizer manufacture (5, 6). This source alone produces an estimated 300 million pounds of triuret annually that is applied on agricultural lands worldwide (7). Thus, triuret is an additional source of nitrogen that can, upon degradation, support microbial growth. Engelhardt *et al*. used this concept to isolate a *Corynebacterium* species growing on triuret, but no enzymes had been identified prior to this study (8).

A similar compound to triuret, biuret (carbamylurea) with one less carbamoyl group, has a known biodegradation pathway through biuret hydrolase (E.C 3.5.1.84) (9, 10). Biuret hydrolase (BiuH) hydrolyzes biuret to allophanate and is part of the Isochorismatase-like hydrolase (IHL) protein family within the cysteine hydrolase superfamily (10, 11). Allophanate is then hydrolyzed by allophanate hydrolase (AtzF, E.C 3.5.1.54), an amidase, and completes the biuret metabolic pathway. Prior to this study, the only known precursor of biuret was cyanuric acid, a metabolite of *s*-triazine compounds mostly comprised of herbicides (Fig. 1). However, based on bioinformatics analysis, biuret metabolism appears to be mostly independent of cyanuric acid metabolism (9, 10). In lieu of the cyanuric acid hydrolase (AtzD, E.C 3.5.2.15) not being in an operon with BiuH, the genome contexts of several hundred BiuH encoding sequences revealed a co-occurring homologous sequence, TrtA, with ∼50% amino acid sequence identity on average but showing no activity on biuret. Interestingly, the active sites of TrtA and BiuH are almost identical in sequence, and so it was hypothesized that triuret is the substrate for TrtA, funneling into biuret metabolism. In this study, TrtA was tested as a triuret hydrolase and characterized with respect to substrate specificity, steady-state kinetics, X-ray crystallography and compared with BiuH. A particular focus was on the ability of TrtA to discriminate triuret from biuret despite its near-identical active site to BiuH that is highly active with biuret.

**Figure 1:**
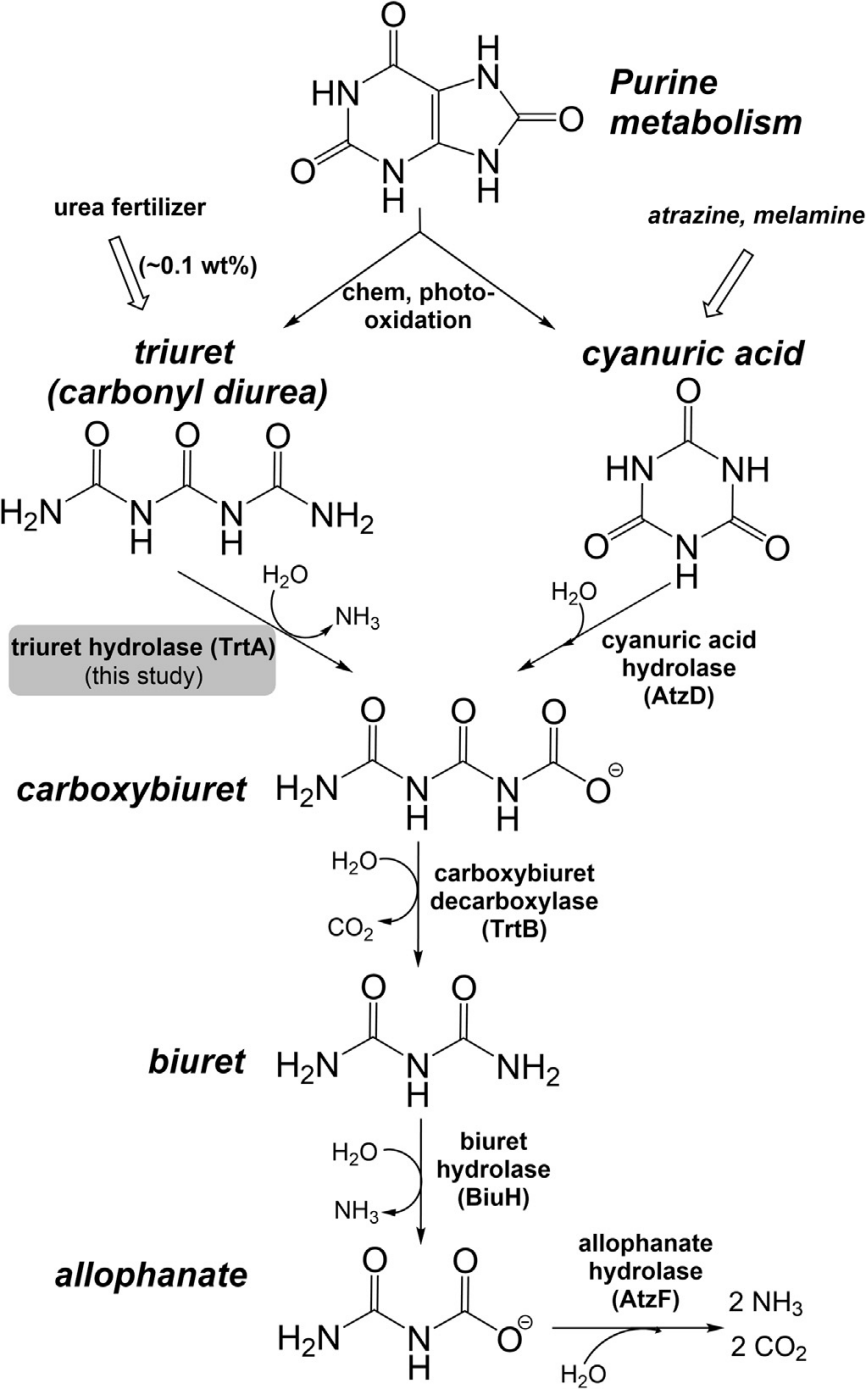
**Triuret and cyanuric acid mineralization pathways through biuret.** Chemical oxidation of uric acid by peroxides, peroxynitrite and photooxidation can yield triuret and cyanuric acid in addition to anthropogenic sources of agricultural nitrogen fertilizer and *s-*triazine pesticides. Triuret and cyanuric acid hydrolase pathways converge through carboxybiuret, which includes enzymes AtzF, an amidase, AtzD, a ring-opening hydrolase, TrtB, a decarboxylase, and BiuH, TrtA homologs in the IHL protein family.

## Results

### TrtA is a triuret hydrolase that hydrolyzes triuret to form carboxybiuret, which feeds into biuret metabolism

The triuret hydrolase (*trtA*) gene (NCBI accession number PKOI01000001.1) tested in this study comes from *Herbaspirillum* sp. BH-1, which was isolated from a biuret-fertilized potato field. The triuret hydrolase enzyme, TrtA, from the *Herbaspirillum* species shows 48% amino acid sequence identity to the previously crystallized BiuH from *Rhizobium leguminosarum b.v viciae* 3841 (NCBI accession no. CAK10578.1) (9, 10). Both species have operons that contain TrtA, BiuH and AtzF that combine to support the growth of the bacteria by liberating all four nitrogens from triuret as assimilable ammonia (Fig. S1). TrtA and BiuH are members of the IHL protein family that possess the catalytic triad comprised of a cysteine, aspartate, and lysine residue (Fig. S2). TrtA has a subunit molecular weight of 24 kDa and a T_m_ of 65 ^o^C (Fig. S3). Michaelis–Menten kinetics were observed for TrtA with k_cat_/K_M_ of 6.1 x 10^5^ M^-1^s^-1^ (Table 1). The pH optimum of the enzyme, where there is maximal activity, was dependent on the cleavage of the his-tag, increasing from pH 6.5 to pH 8 when cleaved. A C-terminal his-tag was used initially, but this form was less active and produced aggregates. Instead an N-terminal His-tag was used in subsequent experiments. To remove a possible intrinsically disordered region, a C-terminal deletion was made for crystallographic purposes. A multiple sequence alignment of the TrtA sequence revealed additional C-terminal residues not common to other TrtA proteins. This deletion mutant had negligible effect on the activity of the enzyme but did increase the expression yield by more than twofold. TrtA is very specific for triuret, no other substrate tested had more than 0.5% of the activity observed for triuret (Table 2). Surprisingly, formylurea and 1-nitrobiuret were more reactive than biuret while nonplanar compounds methylene diurea and succinamide showed no detectable reactivity.

**Table 1:** Kinetic parameters of TrtA constructs

Variant	pH_*opt*_	K_*M*_ (*μ*M)	k_*cat*_ (1/s)	k*_cat_*/K*M* (M^−1^s^−1^)
TrtA	6.5	21 ± 3	11 ± 1	5.3 ± 1.0 × 10^5^
+Cleaved His-tag	8	15 ± 3	8.8 ± 0.4	6.1 ± 1.6 × 10^5^
CtermDelΔ7+Cleaved His-tag	8	16 ± 2	10 ± 1	6.3 ± 1.3 × 10^5^

**Table 2:** Substrate specificity of TrtA

Substrate	Specific activity (U/mg)	Specificity ratio
Triuret	24	1
Formylurea	0.07^a^	2.9 × 10^−3^
1-Nitrobiuret	0.002^a^	8.3 × 10^−5^
Biuret	0.0004	1.7 × 10^−5^
Acetylurea	0.0001	4.2 × 10^−6^
Tetrauret, pentauret	n.d	
Methylene diurea	n.d	
Succinamide	n.d	

a measured by Ammonia Assay Kit; n.d - not detected.

**Figure 2:**
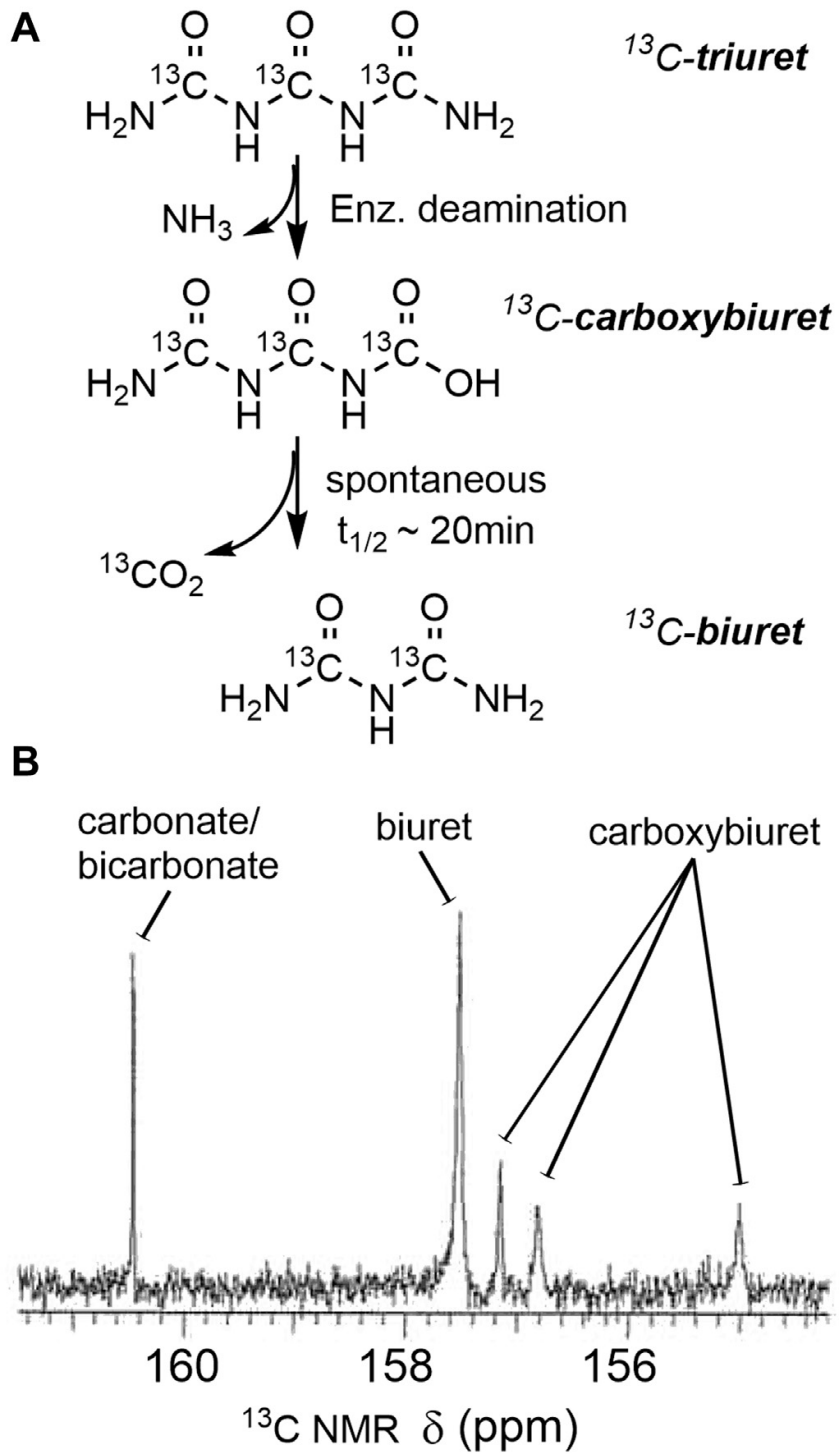
**Elucidation of the reaction product of TrtA by**^**13**^**C NMR.***A*, pathway for the fate of triuret hydrolysis with TrtA to make carboxybiuret and then the spontaneous decarboxylation to form biuret. *B*, ^13^C NMR of labeled triuret hydrolysis by TrtA. ^13^C triuret (∼50 mM) was added to 120 μM TrtA in 125 mM phosphate buffer pH 8 and stirred for a few minutes before doing NMR acquisition, which totaled to a 10-min incubation.

The TrtA reaction produced biuret, as shown by ^13^C NMR, and one ammonia molecule, as measured *via* the Berthelot reaction (Fig. 2*A*). The TrtA reaction could produce ammonia directly or indirectly by cleaving the terminal or subterminal C-N bonds of triuret, respectively. The former reaction pathway was shown to be operative using ^13^C triuret and ^13^C NMR (Fig. 2*B*). After 10 min of reacting ^13^C-triuret and TrtA, three distinctive resonances of carboxybiuret were clearly discernible at 155, 156.8, and 157.1 ppm. Those resonances remained for minutes and then decreased with concomitant increases in biuret and carbonate/bicarbonate. From the time course, we estimated the half-life of carboxybiuret decarboxylation to be ∼20 min, consistent with previous studies with the reaction of cyanuric acid to carboxybiuret (15). In cyanuric acid degrading bacteria, carboxybiuret has been shown to be metabolized by either of two distinct pathways, *via* a decarboxylase or a deaminase (16, 17).

### Crystallization and overall structure of TrtA

**Figure 3:**
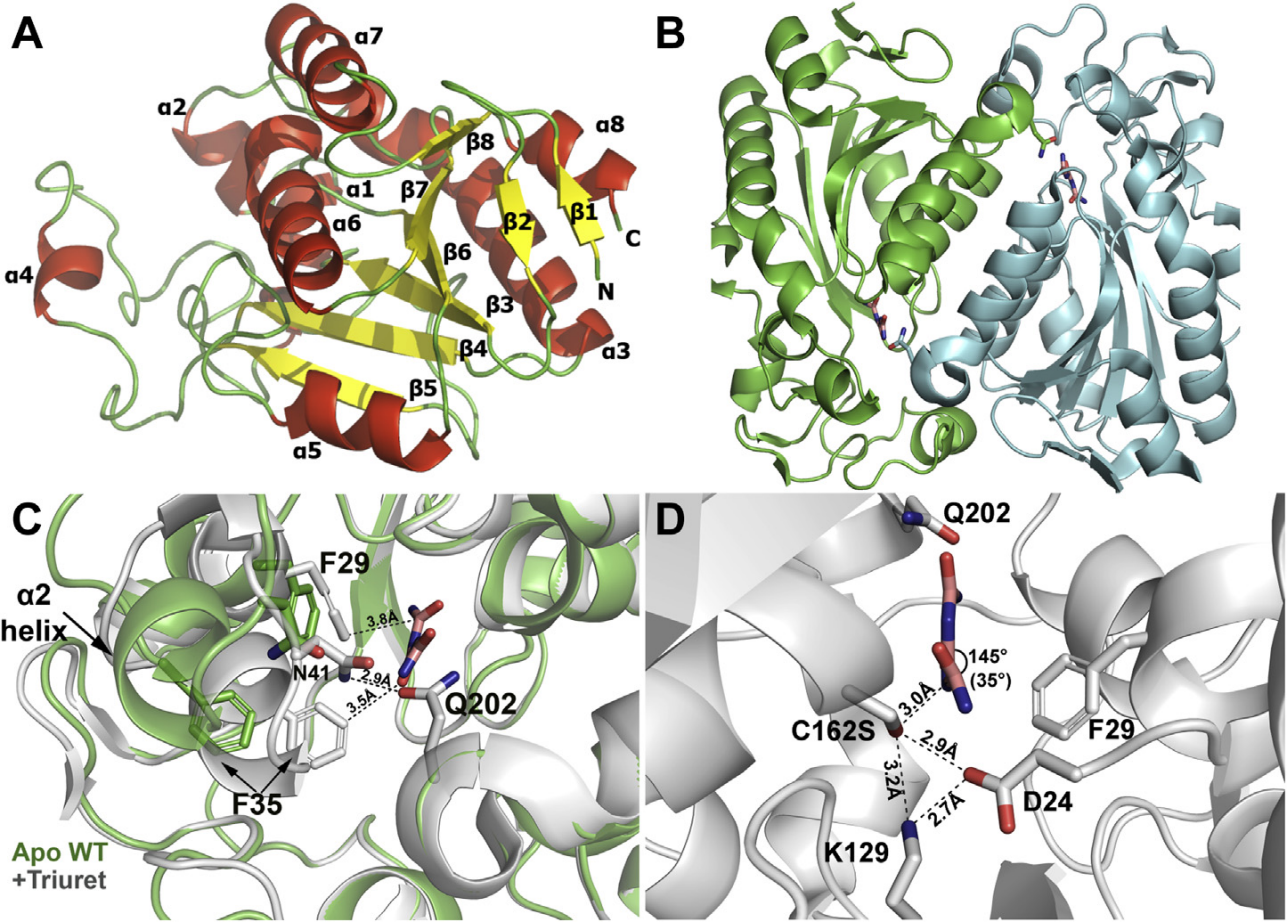
**Topology and open–closed conformations of TrtA.***A*, cartoon representation of a monomer structure of TrtA. Loops are colored green, α helices are in red, and β sheets are in yellow with N- and C-termini-labeled N and C, respectively (PDB 6XIX). *B*, TrtA dimer structure: Cartoon representation of the TrtA dimer with triuret bound in the two active sites, which are complemented by both subunits (PDB 6XJE). *C*, open, closed conformations of TrtA upon substrate binding. The apo WT (PDB 6XIX) and triuret bound (PDB 6XJE) structures of TrtA overlaid show a conformational shift of the α2 helix toward the active site upon substrate binding with notable residues being F29, F35, and N41. *D*, catalytic triad and conformation of triruet bound in active site. The catalytic residues of TrtA are in positioned relatively to each other in a triangle comprised of D24, K129, and C162, which was mutated to serine (C162S) to render TrtA inactive and obtain cocrystal. While triuret is hypothesized to be a planar molecule in solution, when bound in the active site of TrtA, triuret appears to be in a preattack complex where the torsion angle is 35 degrees for the terminal ureide closest to the catalytic residues (PDB 6XJE).

Crystals of TrtA diffracted in the range of 1.4–2.1 Å with the structure solved by molecular replacement using the *Rhizobium* BiuH structure, PDB 6AZO (Table 3). TrtA elutes as a dimer by size-exclusion chromatography, and the monomer adopts a fold typical of the IHL protein family with six parallel β-strands alternating with six α-helices (Rossmann-like) in addition to a two-strand antiparallel β-sheet at the N-terminus (Fig. 3, *A* and *B*) (18). Helices α6 and α7 provide most of the interactions between the two subunits in the dimer while α4 also has some contact at this interface. The α2 helix shows the highest mobility as illustrated by high B-factor values in the apo crystal and undergoes a significant conformational change (∼4.5 Å) when triuret or biuret is bound in the active site (Fig. 3*C*).

**Table 3:** Summary of X-Ray data collection and refinement for *Herbaspirillum* sp.

PDB	6XIX	6XJE	6XJ4
Crystal	WT	C162S + 1 mM Triuret	C162S + 30 mM Biuret
Data collection			
Beamline	24-ID-E	23-ID-D	23-ID-B
Wavelength (*A*˚)	0.979	1.033	0.992
Space group	P1	P2_1_2_1_2_1_	P212121
Dimension a, b, c (*A*˚)	72.95, 76.55, 93.59	51.53, 114.44, 142.93	51.63, 114.18, 141.47
Dimension *α*, *β*, *γ* (^*o*^)	118.40, 89.61, 103.07	90.00	90.00
Resolution (*A*˚)	81.57−2.10 (2.20−2.10)^a^	60.62−1.45 (1.55−1.45)^a^	57.09−1.78 (1.88−1.78)^a^
No. observed refle^a^tions	194240 (26,162)	1121865 (194223)	594818 (88,093)
No. unique reflections	91,216 (12,132)	150183 (26,952)	154547 (23,387)
R_*meas*_ (%)	9.1 (23.8)	5.1 (54.7)	7.2 (43.1)
CC(1/2)	0.991 (0.950)	0.999 (0.877)	0.997 (0.911)
*I*/*σ*	9.16 (4.76)	20.99 (3.97)	12.44 (4.26)
Completeness (%)	90.4 (92.2)	100 (100)	99.8 (99.9)
Redundancy	2.13 (2.16)	7.47 (7.21)	3.85 (3.77)
Refinement			
Resolution (*A*˚)	2.10	1.45	1.78
R_*work*_/R_*free*_	0.1781/0.2267	0.1708/0.1894	0.1637/0.1921
No. atoms			
Protein	13,451	6806	6756
Ligand/Ion	32	48	27
Water	784	407	413
Mean B-factors (*A*˚^2^)	21.9	21.6	29.3
R.m.s deviations bond lengths (*A*˚)	0.009	0.016	0.007
Bond angles (^*o*^)	1.56	2.00	0.91
Ramachandran plot (%)			
Core	97.05	98.58	98.58
Allowed	2.37	0.95	0.95
Dissallowed	0.58	0.47	0.47

BH-1 TrtA.

a Numbers in parentheses refer to the highest-resolution shell.

### Active site of TrtA

**Figure 4:**
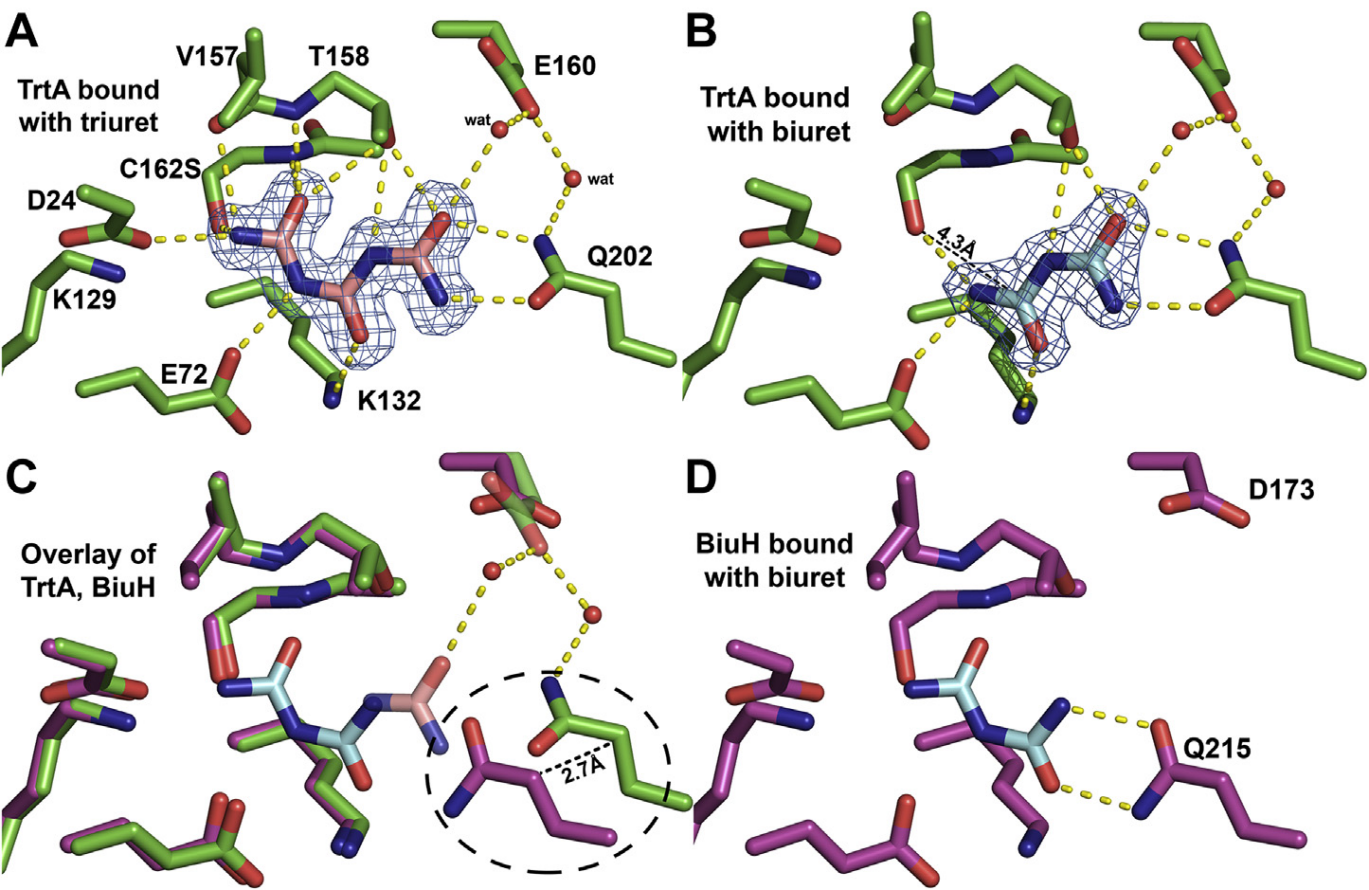
**Overlay of TrtA, BiuH active sites indicates a key glutamine residue.***A*, TrtA bound with triruet. Active site of TrtA with a Fo-Fc omit map contoured at 3σ carved around triuret (PDB 6XJE). *B*, TrtA bound with biuret. Fo-Fc omit map contoured at 3σ carved around biuret bound in the active site of TrtA (PDB 6XJ4). Biuret binds in a noncatalytic fashion in TrtA as compared with active complexes of triuret bound in TrtA and biuret bound in BiuH where the distance between the serine hydroxyl and nearest biuret carbonyl is 4.3 Å, whereas proposed active complexes have this distance at 3 Å. Biuret is a weak inhibitor of triuret hydrolysis and is hypothesized to have a competitive mode of inhibition. *C*, overlay of TrtA, BiuH active sites. Almost all of the active site residues are conserved, and in the same position, except for a glutamine residue. This single residue is the proposed structural determinant of biuret and triuret hydrolysis and is labeled by a dashed circle. *D*, active site of BiuH bound with biuret. Residues Q215 and D173 are labeled to highlight potential key structural differences in TrtA and BiuH active sites (PDB 6AZQ). Select polar contacts made with ligands are marked by yellow dashes.

The catalytic triad that has been previously defined in the cysteine hydrolase superfamily is present in TrtA. It is represented by residues D24, K129, and C162 (Fig. 3*D*). Also present in TrtA is a *cis*-peptide bond in the active site between residues V157 and T158, which is signature of the IHL protein family (Fig. 4*A*) (18). The active site is complemented by residues from the other subunit in the dimer, specifically Q202 that comes from the α7 helix, which coordinates biuret and triuret in bound structures (Figs. 4, *A* and *B*). In the apo WT structure, *beta*-mercaptoethanol (BME) was found covalently attached to the catalytic cysteine of every subunit (Fig. S4). The enzyme was rendered unreactive by a C162S mutation, and this allowed for examining the occupancy of the active site with biuret and triuret. In the triuret cocrystal, with all four active sites occupied, the substrate makes several hydrogen bonds with residues D24, E72, K132, and Q202 from the other subunit in addition to making hydrogen bonds with the backbone amide from the *cis*-peptide bond (Fig. 4*A*). Triuret also has a polar contact with a water molecule coordinated by T158 and E160, and the latter residue also coordinates another water molecule with Q202. The planarity of the triuret in the active site is distorted as the terminal ureide closest to the catalytic residues is bent with an average torsion angle of 35^o^ while the other torsion is fairly planar, 4.5^o^ on average (Fig. 3*D*). This appears to be a preattack conformation, and triuret in this crystal is in a different conformation compared with the crystal structure of pure triuret (Fig. S5). In the active site of TrtA, triuret appears to be in a *cis* conformation that allows for an intramolecular H-bond from a middle nitrogen to the terminal carbonyl closest to the catalytic residues. This is not observed in the crystal structure of pure triuret where it is in the *trans* conformation, and intramolecular hydrogen bonds are from the terminal amines to the central carbonyl (19). B-factor analysis of the triuret substrate in TrtA indicates that the terminal nitrogen atom, closest to the catalytic residues, exhibits the most thermal motion compared with the rest of the triuret atoms (Fig. S5*C*). Anisotropic B-factors of the TrtA-triuret complex show no preferential direction for movement of the active-site loops.

### Open–closed conformations of TrtA

The α2 helix (residues 35–39) undergoes a conformational change when bound with biuret or triuret (Fig. 3*C*). The closed conformation is observed in all of the chains of the triuret cocrystal as well as for a single chain in the biuret cocrystal, which has biuret bound. The other chains in the biuret cocrystal that do not have biuret bound, as well as the apo WT crystal, are in the open conformation. Two residues, F35 and N41, from the α2 helix make significant conformational changes from the open to closed state where F35 moves ∼ 5 Å to make van der Waals (VDW) interactions with the substrate and N41 moving into hydrogen bonding distance with Q202 (Fig. 3*C*). Another residue that changes conformation slightly is F29, which closes in toward the substrate by VDW interactions. This phenomenon of open-closed conformations is present in BiuH but has not been reported in any other members of the IHL protein family (10, 18, 20, 21).

### Comparison of TrtA and BiuH structures

The structure and sequence of TrtA are comparable with the biuret hydrolase structure but different in several important ways. The key differences are the loss of the two-strand β-sheet (β1 and β2) at the N-terminus and the elongation of the α4 helix in the biuret hydrolase (Fig. S6). In the active site, almost all of the residues are conserved between TrtA and BiuH with the major difference being the position of the Q202 residue. In BiuH, Q215 is farther inside the active site by ∼2.7 Å (Fig. 4*C*). Triuret and biuret bound in their respective enzymes align in a similar manner. They each make similar dihedral angles and the same hydrogen bonds while triuret has an additional polar contact with a water molecule coordinated by E160 residue. In BiuH, the corresponding residue is an aspartate (D173), and waters coordinated by this residue are not in the same positions as in TrtA and do not have interactions with the substrate in BiuH.

### Biuret inhibition of TrtA

Biuret shows very low activity and is a very weak inhibitor of TrtA (Table 2). The cocrystal containing biuret, at a concentration of 30 mM biuret, only occupied one of four active sites in the unit cell. Biuret is coordinated by a subset of the residues that coordinate triuret, and it binds in a noncatalytic fashion (Fig. 4*B*). The dihedral angle for biuret is fairly planar in this structure and binds in the same place where triuret is planar as well. Inhibition data suggests that biuret is a competitive inhibitor. However, since biuret contains a 1% (mol) impurity of triuret, the K_i_ cannot be determined due to the high biuret concentrations required. Substantial inhibition is only observed at concentrations of biuret greater than 50 mM. Also, at those concentrations, TrtA precipitates due to the chaotropic properties of biuret (Fig. S7). Attempts to crystallize active WT TrtA in biuret solutions led to the formation of very small crystals and soaking experiments with biuret resulted in cracking of the crystals. However, the inactive mutant C162S was able to produce large cocrystals with biuret (Fig. 4*B*).

### Active site mutagenesis of TrtA

**Figure 5:**
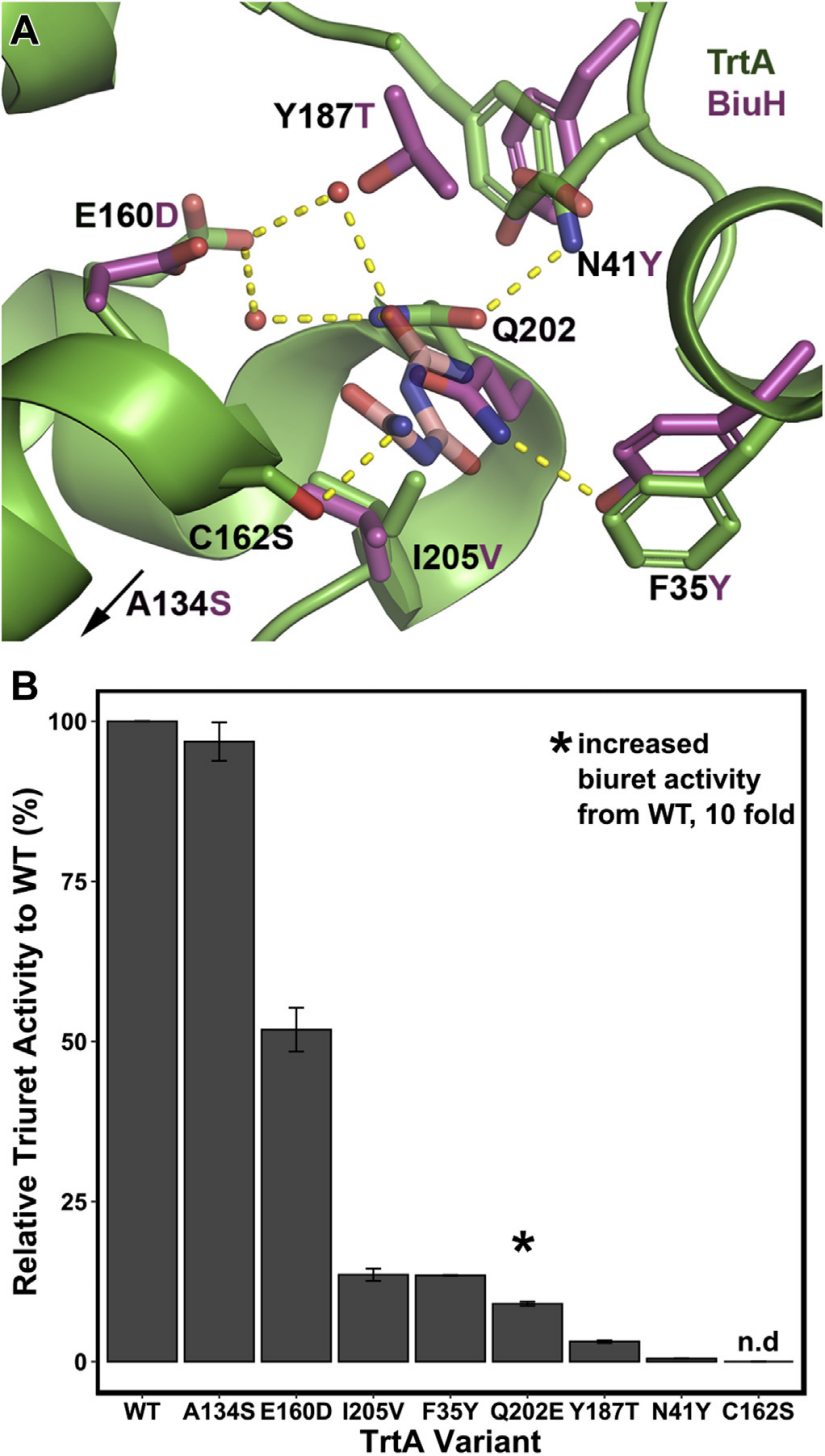
**Mutagenesis of TrtA consensus residues to BiuH consensus.***A*, active site mutagenesis of TrtA. Select TrtA residues are shown with the BiuH consensus at those positions as determined by multiple sequence alignment of over 500 TrtA and BiuH sequences (PDB 6XJE). The C162 and Q202 were mutated to serine and glutamate, respectively, to probe their role in triuret hydrolysis. The A134 position in TrtA is ∼7 Å behind the C162 position away from the active site and showed high sequence conservation in TrtA as an alanine and in BiuH, a serine residue. Select polar contacts are marked by yellow dashes. *B*, triuret activity of TrtA variants relative to WT. The majority of variants were deleterious to triuret activity except for the A134S variant. The C162S variant used to cocrystallize TrtA with triuret and biuret had no activity and hence is labeled with n.d (not detected). The Q202 E variant lost >95% of its triuret activity but increased tenfold in biuret activity where no other variants saw an increase.

Critical activity-determining residues in the active site of BiuH are largely conserved in TrtA (10). Alanine scanning of D36, F41, K142, K145, and Q215 in BiuH yielded complete activity loss with exceptions for the phenylalanine and glutamine residues that saw >90% activity reduction (10). The corresponding residues in TrtA are D24, F29, K129, K132, and Q202, respectively. To expand the scope of residues critical for triuret activity and to identify the mechanism of how triuret and biuret are discriminated, select residues were identified from the discrete differences between the sequence motifs of TrtA and BiuH. The positions were F35, N41, E160, Y187, I205, and A134 (Fig. S8). The residues F35, N41, Y187 lie at the entrance to the active site while E160 and I205 interact with Q202 by a H-bond network, for the former, or by VDW interaction for the latter residue (Fig. 5*A*). A134 is positioned behind the catalytic C162 residue in the core of the protein, and while all the residues around this alanine are conserved in both TrtA and BiuH, the alanine alone is by consensus, in BiuH, a serine residue. The mutagenesis strategy was to mutate the residues in TrtA to the corresponding residue in BiuH. We found that the majority of the mutations were deleterious to triuret activity except for A134S (Fig. 5*B*). The variant E160D had 50% of the maximal activity while single variants F35Y, Q202E, Y187T, and N41Y had less than 10%. As mentioned previously, the C162S variant was inactive and used to cocrystallize TrtA with triuret and biuret. No single mutant saw an increase in biuret activity except for Q202 E, which saw a ten fold increase but was still three orders of magnitude less than its activity on triuret.

### Kinetics of a biuret, triuret hydrolyzing generalist

As the mutagenesis experiment could not yield significant improvement of biuret activity in TrtA, the attention became directed at characterizing a naturally occurring enzyme that had generalist properties in hydrolyzing biuret and triuret at similar rates. A generalist enzyme (RhdBiuH), with 67% sequence identity to BiuH, was found in the genome of *Rhodococcus* sp. Mel, which is colocated with a cyanuric acid hydrolase (AtzD) and has no triuret hydrolase in the genome (9). The enzyme hydrolyzes biuret at a relatively slow rate (1.7 U/mg) compared with true BiuHs (>10 U/mg) and has a specific activity for triuret of 0.3 U/mg, which is 25-fold higher than BiuH (Table 4). The K_M_ values for biuret and triuret of this enzyme were more than ten fold higher than for TrtA or BiuH, respectively. Saturation kinetics were not achieved for RhdBiuH with triuret as the substrate, but the k_cat_/K_M_ could still be measured in the linear regime of Michaelis–Menten kinetics (K_M_ >> [S]). Crystallizing the RhdBiuH enzyme was done in an attempt to see triuret bound in the active site of a BiuH and possibly explain the generalist properties of the enzyme (Table S1). Cocrystals of the inactivated RhdBiuH enzyme (C169S) made with biuret and triuret diffracted at resolutions similar to TrtA crystals. However, despite our efforts, triuret was not bound to the active site, possibly due to low affinity for the substrate (Fig. S9). Instead of triuret, electron density maps show an unidentifiable, tetrahedral adduct attached to the serine mutant (C169S), which was left unmodelled (Fig. S9*D*). The cocrystal with biuret showed similar mode of binding to that of BiuH and identical active site residues (Fig. S9*C*). The open–closed phenomenon was not observed in the RhdBiuH, but this may be due to the closed conformation facilitating crystal formation (Fig. S9*A*).

**Table 4:** Kinetic parameters of BiuHs for biuret and triuret

Enzyme	Substrate	K_*M*_ (_*μ*_M)	k_*cat*_ (1/s)	k_*cat*_/K_*M*_ (M^−1^s^−1^)	Note
RhdBiuH	Biuret	95 ± 26	1.5 ± 0.1	7.0 ± 2.0 × 10^3^	
	Triuret	>1000	0.14^a^	1.7 ± 0.4 × 10^2^	
BiuH	Biuret	23 ± 4	4.0 ± 0.2	1.7 ± 0.3 × 10^5^	Cameron *et. al*
	Triuret	>1000	0.0044^a^	n.m	n.m - not measured

a Saturation kinetics were not achieved and activity was measured at 1 mM triuret RhdBiuH—*Rhodococcus* sp. Mel BiuH; BiuH—*Rhizobium leguminosarum* b.v 3841 BiuH.

## Discussion

### TrtA poses an interesting evolutionary question in that it is very specific for triuret; yet the activity on biuret is four orders of magnitude less than on triuret

TrtA has a catalytic efficiency on the order of 10^5^ M^−1^s^−1^, which is typical for enzymes active on natural metabolites (Table 1) (22). In addition, TrtA displays exquisite substrate specificity for triuret with other substrates only having several orders of magnitude less activity than for triuret (Table 2). Discriminating against small substrate molecules is an evolutionary challenge, which often requires different structural folds if discrimination between substrates cannot be achieved in the same fold (23). An amidase, not in the IHL protein family, already exists to hydrolyze carboxybiuret, the TrtA reaction product, to dicarboxyurea, which is named AtzEG and found in the cyanuric acid mineralization pathway of *Pseudomonas* sp. ADP (17). The enzyme AtzE and AtzF, the latter being an allophanate hydrolase, have a similar relationship like BiuH and TrtA. AtzE and AtzF are homologs of each other and their native substrates differ by only one additional amide group, although in this case, the active site residues coordinating the substrates are mostly different (17, 24, 25). TrtA is not the only enzyme to generate carboxybiuret. Cyanuric acid hydrolase (AtzD) also produces carboxybiuret through opening of the ring, and a ^13^C NMR study of this reaction produced very similar results to TrtA by observing carboxybiuret, biuret, and bicarbonate/carbonate peaks (15). The spontaneous decarboxylation of carboxybiuret to biuret that is observed *in vitro* can be actually enzyme catalyzed in microorganisms that possess the recently discovered decarboxylase gene *trtB*, which co-occurs with TrtA and AtzD in genomic contexts (16).

### Discrimination of triuret versus biuret: the role of Q202

While TrtA and BiuH have the same residues to coordinate triuret and biuret, the key difference between the two is the position of the Q202 residue. In BiuH, it is closer to coordinate the smaller biuret, and in TrtA, it is farther out to accommodate triuret (Fig. 4*C*). The biuret cocrystallized with TrtA binding in a noncatalytic fashion suggesting that the glutamine residue has a strong role in binding the substrate and stabilizing the preattack conformation as biuret is fairly planar in this structure (Fig. 4*B*). Interestingly, Q202 in TrtA does not change conformation in either the open or closed conformations despite it being in a different position in BiuH (Fig. 3*C*). Therefore, it is possible that changes in second shell residues around this glutamine serve to switch the enzyme from a triuret to a biuret hydrolase (Fig. 4*C* and 5*A*). The mutation at E160 to aspartate removed the coordination of the water molecules that hydrogen bond with triuret and Q202 and led to a 50% loss in activity. Mutations of residues related to the open–closed conformational transition, namely N41Y and F35Y, were deleterious to triuret activity perhaps affecting the mobility of the α2 helix, which may be crucial in substrate tunneling and product release (Figs. 3*C* and 5*A*). The residue I205 in TrtA prohibits, by steric hindrance, Q202 from being able to move more inside the active site, in a conformation similar to that observed in BiuH. The residues that coordinate the Q202 residue at its current position in TrtA are N41, by hydrogen bonding and *via* a water molecule, E160, where mutation of these latter two residues saw deleterious effects to triuret activity (Fig. 5). Substituting Q202 in TrtA with a glutamate residue disrupted the hydrogen bonds that could be made with triuret and biuret, and as a consequence there was significant activity loss for triuret hydrolysis but a tenfold increase in biuret reactivity. This may be attributed to the disruption of the inhibitory binding mode of biuret and as a result promoting the mode of binding for biuret to act as a substrate (Figs. 4, *B* and 5).

The selectivity of TrtA, favoring triuret heavily, could have a physiologic importance in that biuret is inhibitory to the enzyme, and this provided the evolutionary pressure for TrtA to achieve greater selectivity (*i.e.,* a counter selection). Alternatively, the selectivity could be a consequence of being catalytically capable of hydrolyzing either biuret or triuret. But both triuret and biuret may require proper prealignment for productive bindings, and this may be consistent with the discrete conformation of Q202 in TrtA independent of the active site loop movements. In BiuH, N41 is a tyrosine (Y53) that may sterically hinder the glutamine from moving away from the active site, like in TrtA, but with a valine residue at I205 (V218), it can be as close as it is in the active site for which the glutamine residue is then fixated by another tyrosine at the TrtA position of F35 (Fig. 5*A*).

**Figure 6:**
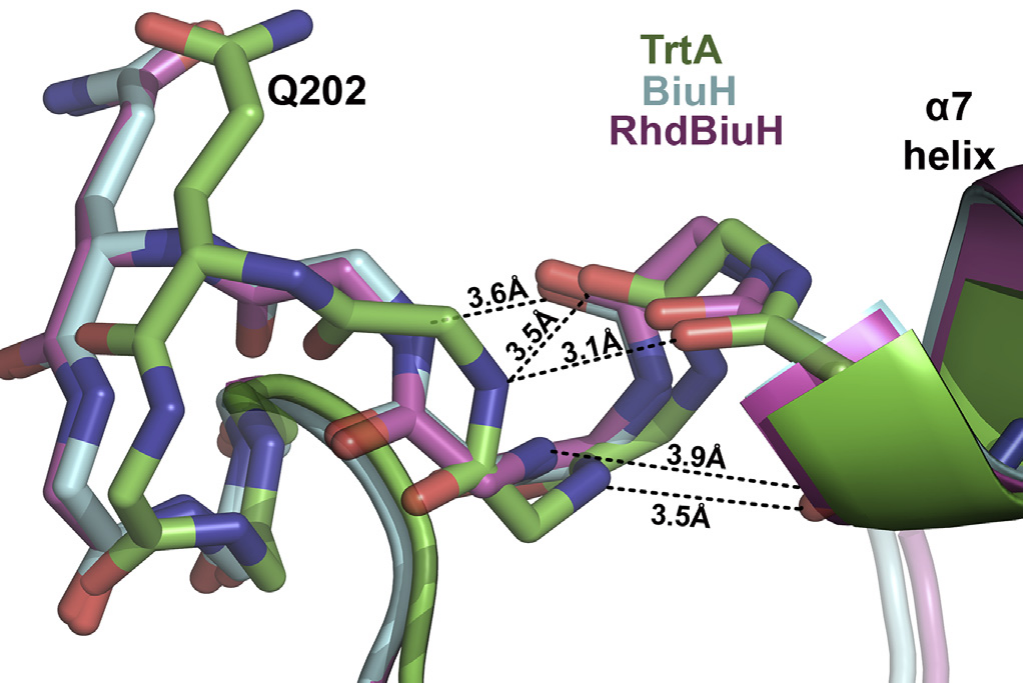
**Backbone conformation of critical glutamine residue dictates biuret, triuret specificity.** Overlay of the backbone of TrtA (PDB 6XJE), BiuH (PDB 6AZQ), and RhdBiuH (PDB 6XJM) around the critical glutamine residue (Q202). The loop of TrtA, in this region, is in a different conformation than BiuH and RhdBiuH possibly due to more H-bond networks with the preceding α7 helix.

The exact way the position of the critical glutamine residue (Q202) has evolved to specialize in biuret and triuret activity is still unknown. The oligomeric state where biuret hydrolases are tetrameric and triuret hydrolases are dimeric does not appear to play a role, where the dimer–dimer interface is >20 Å to the critical glutamine residue. While no single mutations reported here served to increase TrtA’s biuret activity, it is likely that a combination is required for the evolution of a biuret hydrolase from TrtA and vice versa. A key question is how the backbone conformation of the loop, which harbors the critical glutamine residue, comes to be. As seen in Figure 6, it appears that the secondary structure differs in TrtA and BiuH in this area where the backbone atoms of the preceding residues to the glutamine have intermolecular interactions, in TrtA, with the α7 helix, whereas in BiuH these interactions are absent. In conclusion, it seems that the fixation of Q202, in addition to its range of motion, is crucial for activity and specificity as it may play a role in stabilizing the preattack conformation of biuret/triuret, where the planarity is perturbed for molecules that should have complete delocalization of electrons.

### Promiscuity of RhdBiuH

The promiscuous activity of RhdBiuH on both triuret and biuret sparks curiosity as observed structural differences between RhdBiuH and BiuH in the active site are minimal despite the differences in substrate specificity between the two enzymes (Table 4, Fig. S9*C*). Arguably, protein dynamics, which are not captured by crystallography, may play a role in these specificity differences that could pertain to the catalytic mechanism and substrate tunneling/product release by the enzyme. In organisms that contain only one copy of a *biuH* gene, like the *Rhodoccocus* sp. Mel species that encodes RhdBiuH, generalist properties may be selected for if they do not possess a *trtA* gene with specialization of certain BiuHs and TrtAs occurring in genomes encoding both.

### Catalytic mechanism of TrtA

In the IHL protein family, the catalytic triad is positioned nonlinearly, unlike other triads.

**Figure 7:**
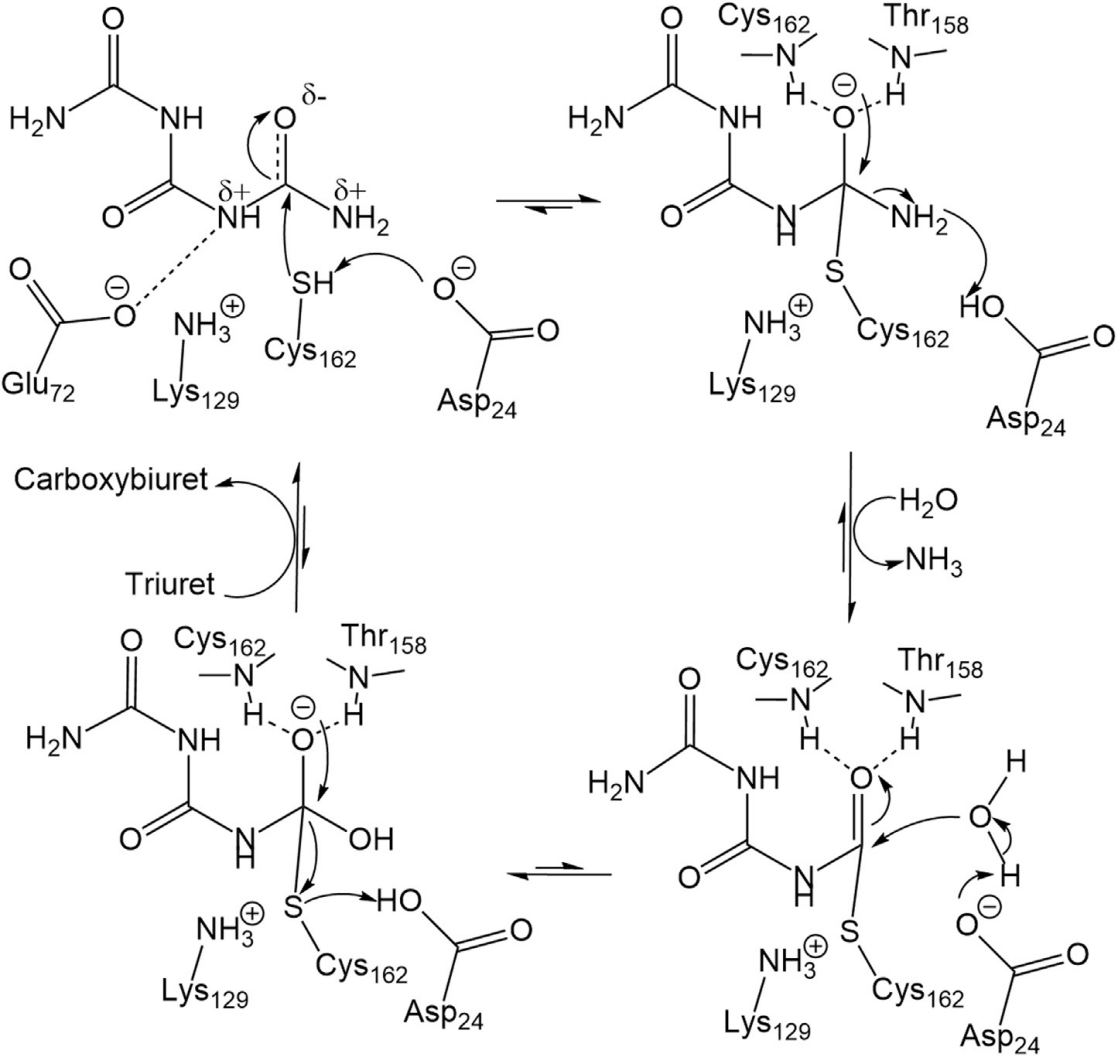
**Mechanism of TrtA. The catalytic triad comprises Asp24, Lys129, and Cys162, signature of the IHL protein family.** The Michaelis complex is proposed to have triuret in a preattack conformation where the carbonyl has lost some sp2 character as observed in the crystal structure. The oxyanion hole is represented by the backbone nitrogen atoms of Cys162 and Thr158.

Instead, catalytic triads of IHL enzymes form a more triangular positioning where the acidic aspartate (D24) helps stabilize the basic K129 but also binds the substrate (Figs. 3*D* and 6) (20, 21). While the catalytic lysine or aspartate can abstract a proton from the catalytic cysteine residue (C162), it is the positive charge of the lysine residue that will effectively lower the pK_a_ of the cysteine and make the residue a stronger nucleophile when negatively charged. The substitution of the cysteine to serine renders the enzyme inactive, potentially due to the serine hydroxyl with a much higher pK_a_ (4–5 units more) than the cysteine thiol (26, 27). The loss of planarity of the triuret for the amide moiety near the catalytic triad suggests that this is a preattack conformation that is made possible by the negatively charged D24 and E72 residues, which help create a dipole moment in line with the carbonyl and make the amide have more sp3 character like the transition state (Fig. 4, *A* and 7). When the cysteine nucleophile attacks to make the tetrahedral transition state, the oxyanion hole is formed by the backbone nitrogen atoms of T158 and from the catalytic cysteine, C162. The amine then leaves as ammonia following the formation of the thioester linkage, which subsequently is attacked by water, proposed to be activated by D24, to make the second tetrahedral transition state (20, 21). The active site is then regenerated when carboxybiuret is released with the catalytic cysteine as the leaving group.

### Biological relevance of triuret

With the discovery of an ultraspecific triuret hydrolase in nature, it could be of interest to investigate the biological significance of triuret in the microbiome and its potential applications. Bioinformatics on the prevalence of *trtA* in recorded genomes suggests that triuret metabolism is relatively rare in recorded bacterial genomes (1–2%) while also present in some fungi and phytoplankton (9, 28). Triuret metabolic genes interestingly come from organisms with genera synonymous with plant-associated bacteria and fungi (mycorrhizal). A possible connection with triuret and plants is the role of uric acid metabolism as the main storage of nitrogen in plants and in pathogen defense (29, 30, 31). In plant nodules, uric acid is formed from the assimilation of nitrogen produced by bacterial endosymbionts fixing atmospheric nitrogen (N_2_), which is then transported through the rest of the plant as allantoin, a uric acid metabolite (32, 33). Xanthine oxidation, which yields uric acid, generates reactive oxygen species, superoxide and peroxide, which serve as a stress and pathogen defense response (31). Reactive oxygen species in plants, superoxide and nitric oxide, can also form peroxynitrite, a known oxidant of uric acid to produce triuret as the sole product (3, 13, 34). The high flux of uric acid in the plant environment could generate triuret as a minor product by oxidation and, most likely nonenzymatically, which plant-associated bacteria and fungi could mineralize with the triuret biodegradation pathway illuminated by this study.

A potential application arises with the existence of the triuret hydrolase in nature. As triuret has low solubility, and the enzymes that metabolize it are relatively rare, triuret may be proposed as a slow-release nitrogen fertilizer for agriculture that could ameliorate the nitrogen pollution caused by contemporary practices of fertilizing agricultural lands (6, 7, 14).

## Experimental procedures

### Molecular cloning and mutagenesis of trtA gene

The triuret hydrolase (TrtA) gene from *Herbaspirillum* sp: BH-1 (NCBI accession no. WP_102661291.1) was codon-optimized and cloned with a N-terminal 6x HisTag and thrombin cleavage site into a pET28a vector using NdeI and HindIII restriction sites and transformed into BL21DE3 *E. coli* cells (New England Biolabs) (9). Site-directed mutants and a C-terminal Δ7 deletion were made using the Q5 Site Directed Mutagenesis Kit from New England Biolabs.

### Heterologous expression of TrtA, purification, and his-tag cleavage

The *trtA* gene was expressed by growing cells in lysogeny broth (LB) medium with 50 μg/ml kanamycin at 37 ^o^C and 200 rpm to an OD_600_ of 0.6 in a shake flask. The culture was cooled to 16 ^o^C and induced with 0.5 mM isopropyl β-D-1-thiogalactopyranoside (IPTG) and, with the same agitation, incubated for 20 h. Cell pellets were harvested by centrifugation at 1500 x g for 20 min and then resuspended in lysis buffer (20 mM sodium phosphate, 0.5 M sodium chloride, 10 mM *beta*-mercapatoethanol pH 7.4). The cells were lysed using a French Press with three passes at 10,000 psi, and the lysate then clarified by centrifugation at 20,000 x g for 1 h.

The triuret hydrolase was purified from the lysate by using fast protein liquid chromatography (FPLC) and Ni-NTA chromatography. Using a GE-AKTA FPLC and a GE HisTrap 5 ml column, TrtA was purified after running an imidazole gradient from 100 mM to 500 mM and fractions were collected. The expression yield for TrtA was 30 mg per liter culture while for a C-terminal deletion mutant, the yield was 70 mg per liter culture. Pooled fractions were either buffer exchanged into buffer containing 20 mM sodium phosphate, 200 mM sodium chloride, and 10 mM *beta*-mercaptoethanol at pH 8 for kinetic experiments or into 50 mM Tris, 200 mM sodium chloride, and 10 mM *beta*-mercaptoethanol at pH 8 for thrombin cleavage and crystallization experiments using a 15-mL Amicon 10 kDa Centrifugal filter. TrtA protein concentration was determined using the Bradford method (Bio-Rad Protein Assay). The His-tag was cleaved from TrtA using bovine thrombin protease (Sigma) by adding two units of thrombin per mg TrtA in a dilute protein solution between 5 and 10 mg/ml TrtA with Tris buffer and the cleavage reaction was placed on a rotator at 4 ^o^C for >36 h. The reaction was concentrated to 2 ml and cleaved TrtA was purified by size-exclusion chromatography using the AKTA FPLC and a GE Healthcare HiLoad 16/600 Superdex 200 pg column. The column was equilibrated with 50 mM Tris 200 mM sodium chloride at pH 8, the sample was injected onto the column and washed with 1 column volume at 1 ml/min flow rate. TrtA eluted as a homodimer at ∼48 kDa and the preparation was then subsequently used in crystallization experiments.

### Crystallization of TrtA

Initial crystallization conditions were found from the sparse matrix screen PEGRx HT (Hampton Research) using 20 mg/ml TrtA. Crystals were grown by vapor diffusion in a 24-well hanging drop crystallization plate, crystal growth was sensitive to changes to the relative humidity. In order to prevent condensation, the air was purged from the wells with compressed nitrogen prior to sealing each well. Crystals grew in a range of conditions at 18 ^o^C between 20 and 30% (wt/v) PEG 6000 and 0.1 M Bis-Tris propane pH 7.5–8.5 in drops of 1 μl of protein (10–20 mg/ml) with 1 or 2 μl of precipitant. Crystals appeared after 1 day as orthorhombic crystals and were harvested by looping them into cryoprotectant (mother solution containing 25% (v/v) ethylene glycol) and frozen in liquid nitrogen.

### X-ray diffraction data collection and structural determination of TrtA

Diffraction data was collected using the Advanced Photon Source (Argonne, Illinois, USA) with various beamlines (Table 3). Data was processed using XDS (Build January 26, 2018) and molecular replacement, refinement was done using Molrep and Refmac within CCP4 (Version 7.0) and Coot (v0.8.9) (35, 36, 37). For molecular replacement, the structure of the biuret hydrolase from *R. leguminosarum* b.v viciae 3841 was used (PDB 6AZO) with 48% sequence identity (10).

Crystals of TrtA showed a maximum size of 0.4 x 0.1 x 0.05 mm and were either in the space group P1 for the apo form or P2_1_2_1_2_1_ for cocrystals with triuret or biuret. (Table 3). The apo form made small, fragile crystals on average while the unreactive TrtA mutant (C162S) cocrystallized with triuret or biuret made larger, more robust crystals for diffraction with resolution between 1.4 and 2.1 Å. The crystals may diffract to slightly higher resolution, though higher-resolution data shells are incomplete and, to be conservative, were not included. In the asymmetric unit of the apo crystal, with P1 space group, there are four homodimers present. The cocrystals, both with space group P2_1_2_1_2_1_ and the same unit cell parameters, have two homodimers in the asymmetric unit. During refinement, default planarity restraints were used initially for triuret and biuret in structures although difference density was still observed and, as a result, the restraints were relaxed to fit the ligands better into the electron density map.

### Enzyme kinetics

Rates of substrate hydrolysis were determined by ammonia release *via* the Berthelot Assay (38). The Berthelot reagent had solution A (1% (w/w) phenol 50 mg/L sodium nitroprusside), which was added to sample containing ammonium, and then solution B (0.5% (w/w) sodium hydroxide, 0.89% (v/v) bleach) was added after to produce a color response, and absorbance was measured at 630 nm. If a substrate interfered with the Berthelot reagent, the Ammonia Assay Kit (Sigma) was used, which involved adding glutamate dehydrogenase and measuring NADPH disappearance by absorbance at 340 nm with α-ketoglutarate being reductively aminated to form glutamate. Enzyme assays were done in triplicate in phosphate buffer at 25 ^o^C. TrtA enzyme concentrations used in the assay were between 0.01 μg/ml and 50 μg/ml to measure substrate hydrolysis with the rate being linearly dependent on enzyme concentration in this range. Negative controls for the assays included no-enzyme and enzyme with no substrate that resulted in an unchanging amount of ammonium in the assay over time. One unit of activity (U) was defined as 1 μmol substrate per minute at the enzyme's pH optimum at 25 ^o^C.

### Circular dichroism and ^13^C NMR experiments

Circular dichroism (CD) was used to determine the T_M_ of TrtA with the Jasco J-815 CD with a 100 μM sample in 20 mM sodium phosphate, 0.2 M sodium chloride pH 8 (Fig. S3). The temperature gradient was from 55 to 90 ^o^C at a rate of 1 ^o^C/min and the wavelength set to 220 nm. ^13^C-NMR experiments using ^13^C labeled triuret were conducted using the Varian Unity Innova 400 and VnmrQ 2.2 software.

### Synthesis of triuret and substrate analogs

Biuret (Sigma), 1-nitrobiuret (Sigma), formylurea (Acros), acetylurea (Alfa Aesar) were all obtained with high purity (>97%). As triuret is an impurity of biuret (<3% by weight), HPLC was used to quantify the concentration of triuret in biuret solutions using a C18 reversed phase method as developed by Kim *et al* (3).

Triuret: Pure triuret was obtained by oxidation of uric acid with hydrogen peroxide in aqueous ammonia as developed previously by Venable *et al* with an ∼10% yield (12). Melting point 236 ^o^C, ^1^H NMR (400 MHz, dmso-d^6^): δ 6.90 (br, 2H), 7.25 (br, 2H), 9.65 (br, 2H).

^13^C labeled Triuret: ^13^C-triuret was synthesized from ^13^C-urea (Sigma) and hydrogen chloride as described in the patent from Scholven Chemie (39). ^13^C-NMR (100 MHz, dmso-d6): δ 152.6, 153.8

Methylene diurea: Methylene diurea was synthesized from urea and formaldehyde as developed by Murray *et al* (40). It contained a water-insoluble impurity, dimethylene triurea. ^1^H NMR (400 MHz, dmso-d6): δ 4.2 (t, 3H, J = 1.2 Hz), 5.65 (br, 4H), 6.48 (br, 2H).

Ethylidene diurea: Ethylidene diurea was synthesized from urea and acetaldehyde as described by Ogata *et al* (41). ^1^H NMR (400 MHz, dmso-d6): δ 1.25 (d, 3H, J = 1.2 Hz), 5.0 (m, 1H, J = 1.2 Hz), 5.65 (br, 4H), 6.3 (br, 2H).

Tetrauret and Pentauret: Tetrauret and pentauret were synthesized from chlorosulfonyl isocyanate (Sigma) and biuret or triuret, respectively, in acetonitrile by a procedure described in the patent from Farbwerke Hoechst AG (42). Tetrauret: Melting point (decomposition) 200 ^o^C, 1H NMR (400 MHz, dmso-d6): δ 6.90 (br, 2H), 7.25 (br, 2H), 9.75 (br, 2H), 10.60 (br, 1H). Pentauret: Melting point (decomposition) 210 ^o^C, 1H NMR (dmso-d6): δ 6.90 (br, 2H), 7.25 (br, 2H), 9.70 (br, 2H), 10.60 (br, 2H)

## Data availability

The coordinates and structure factors have been deposited in the Protein Data Bank under accession codes 6XIX, 6XJE, 6XJ4, 6XJM, and 6XK1.

## Conflicts of interest

The authors declare that they have no conflicts of interest with the contents of this article.
