# Supplementary material for: Discovery of an ultraspecific triuret hydrolase (TrtA) establishes the triuret biodegradation pathway
Source: J Biol Chem. 2020 Dec 1;296:100055. doi: 10.1074/jbc.RA120.015631 (PMC7948467; doi:10.1074/jbc.RA120.015631)
Supplement: Tables and Figures [file mmc1.docx]

Discovery of an ultra-specific triuret hydrolase (TrtA) illuminates the triuret biodegradation pathway

**Lambros J. Tassoulas^1,2^, Mikael H. Elias^1,2^, Lawrence P. Wackett^1,2,*^**

From the ^1^Department of Biochemistry, Biophysics & Molecular Biology, University of Minnesota, Minneapolis, MN 55455; ^2^BioTechnology Institute, University of Minnesota, St. Paul, MN 55108

Running title: *Discovery of an ultra-specific triuret hydrolase, TrtA*

*To whom correspondence should be addressed: Lawrence P. Wackett: Department of Biochemistry, Biophysics & Molecular Biology, University of Minnesota, Minneapolis, MN 55455; [wacke003@umn.edu](mailto:wacke003@umn.edu); Tel. 612-625-3785 ; Fax. 612-625-5780

**Keywords:** TrtA, triuret, biuret, BiuH, substrate discrimination, enzyme evolution, cysteine hydrolase, IHL protein family, nitrogen-containing compound, uric acid oxidation, urea pyrolysis

**SUPPLEMENT**

**Methods**

Biuret hydrolase (BiuH) from *Rhodococcus* sp. Mel (NCBI acc. no. AEX65081.1) was expressed and purified as done with TrtA with a N-terminal His-tag which was cleaved prior to crystallization (See Experimental Procedures). Crystals were grown at 18^o^C by vapor diffusion in a 24-well hanging drop crystallization plate in a range of conditions between 18-24% (wt/v) PEG 3350 and 0.2 M MgCl_2_ pH 5-6.5 in drops of 1μL of protein (5-10 mg/mL) with 1 or 2μL of precipitant. Crystals appeared after one day as orthorhombic crystals and were harvested by looping them into cryoprotectant (mother solution containing 25% (v/v) ethylene glycol) and frozen in liquid nitrogen.

**TABLES**

Supp. Table 1: Summary of X-Ray Data Collection and Refinement for *Rhodococcus* sp. Mel BiuH


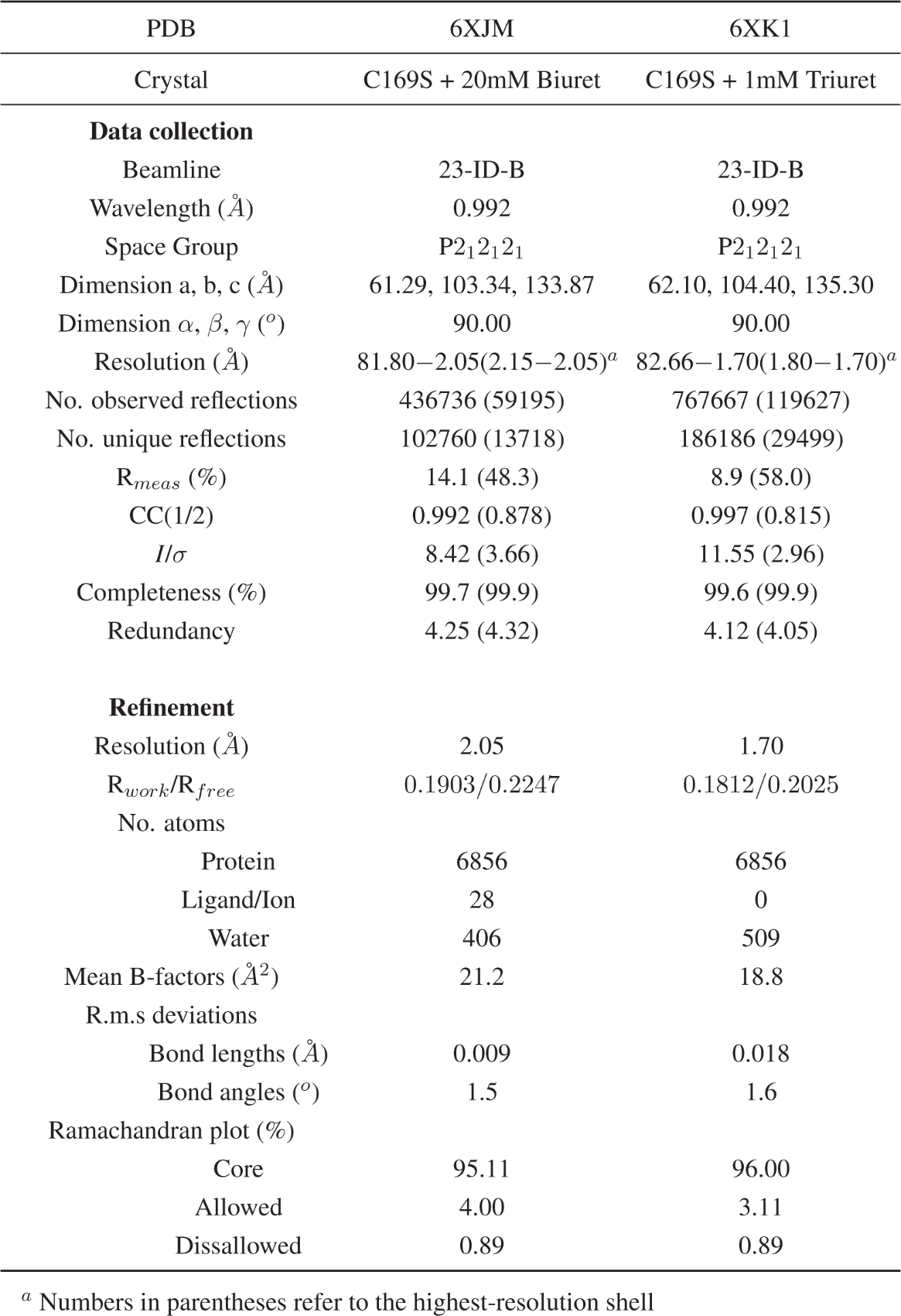


**FIGURE LEGENDS**

**Supp. Figure 1: Gene contexts of *trtA* and *biuH* characterized in the study.** Genomic contexts around *trtA* in *Herbaspirillum* sp. BH-1 and *Rhizobium leguminosarum* bv. viciae 3841 and *biuH* in *Rhodococcus* sp. Mel showing relevant genes to triuret and biuret metabolism including carboxybiuret decarboxylase (*trtB*), cyanuric acid hydrolase (*atzD*), allophanate hydrolase (*atzF*), putative GntR family transcription regulator and ABC transporter cassette. Genes colored in gray encode proteins of unknown function.

**Supp. Figure 2: Multiple Sequence Alignment of TrtA with BiuH and related IHL Protein Family Members:** Sequences are named by PDB ID and aligned using ClustalΩ. Shading of residues indicates sequence conservation and the catalytic triad is highlighted in red. Secondary structure annotation denoted for TrtA.

**Supp. Figure 3: Circular Dichroism of TrtA to obtain Melting Temperature.** Melting temperature of TrtA with cleaved His-tag was determined by Circular Dichroism and estimated to be approximately 65^o^C. Ellipticity was measured at wavelength 220nm with a 1^o^C/min temperature gradient from 45-80^o^C.

**Supp. Figure 4: β-mercaptoethanol Adduct on Catalytic Cysteine in TrtA Active Site.** 2Fo-Fc map for the BME adduct on C162 of TrtA WT contoured at 1σ (PDB 6XIX). Residue color indicates which chain the residue is from. Polar contacts made with BME are marked by yellow dash.

**Supp. Figure 5: Triuret Configuration in TrtA versus Crystalline Form.** (A) *cis* and *trans* isomers of triuret**.** The *cis*-triuret configuration is observed in triuret bound by TrtA where the *trans*-triruet configuration is observed in pure, crystalline triuret as reported by Carlstrohm et al. (B) Torsion angles of triuret bound in TrtA. Triuret has an average 35^o^ torsion angle for the terminal ureide closest to the catalytic residues with the other torsion angle is on average 4.5^o^ for the other terminal ureide. (C) Anisotropic refinement of the TrtA-triuret complex (PDB 6XJE). Thermal ellipsoids shown for triuret atoms with 50% probability of finding the electron density. Average B-factors are labelled for each atom with emphasis on the B-factor for the nitrogen atom closest to the catalytic residue, C162S, colored in red.

**Supp. Figure 6: TrtA, BiuH Overall Structure Overlay.** Cartoon representation of TrtA (PDB 6XIX) overlaid with BiuH (PDB 5BK6). N and C termini are labelled with arrows.

**Supp. Figure 7: Multiple Sequence Alignment of Several TrtA and BiuH Sequences indicating Conserved Residues.** Sequences are named by genus and putative function and aligned using ClustalΩ. Shading of residues indicates sequence conservation and those shaded red are positions where there is consensus in TrtA sequences for one specific residue and in BiuH there is consensus for another amino acid.

**Supp. Figure 8: Weak inhibition of TrtA by Biuret.** The IC_50_ of biuret for TrtA was estimated by measuring V_max_ of TrtA when varying the biuret concentration and keeping the triuret concentration constant at 500μΜ. Error bars showing 1 standard deviation from the mean. The K_i_ for biuret was then estimated to be between 1-2mM using the Cheng-Prusoff equation and assuming the mode of inhibition to be only competitive with triuret(44). As triuret is an impurity in biuret (1 mol%) the estimation of the K_i_ is given in a range of 1-2mM.

**Supp. Figure 9: RhdBiuH Structure Comparison to TrtA and BiuH.** (A) Overlay of Apo (PDB 6XK1) and Biuret Bound (6XJM) forms of RhdBiuH C169S. Active site of RhdBiuH with Fo-Fc omit map contoured at 3σ carved around biuret. An open conformation as seen in TrtA was not captured in the Apo RhdBiuH crystal structure. (B) Overlay of Apo RhdBiuH (PDB 6XK1) and Apo TrtA (PDB 6XIX). Active site overlay of RhdBiuH and TrtA shows the former is already in the closed conformation where Apo TrtA is in the open conformation. (C) Overlay of Biuret bound RhdBiuH (PDB 6XJM) and BiuH (PDB 6AZQ). The mode of biuret binding is nearly identical in RhdBiuH and BiuH and active site residues are identical in position in both. (D) A Putative tetrahedral adduct bonded to C169S in RhdBiuH co-crystalized with triuret. Active site of RhdBiuH with Fo-Fc map contoured at 3σ. Approximate sigma levels for each of the three peaks in the Fo-Fc map are labelled to show the relative sizes of the peaks.

**Supp. Figure 1**

**
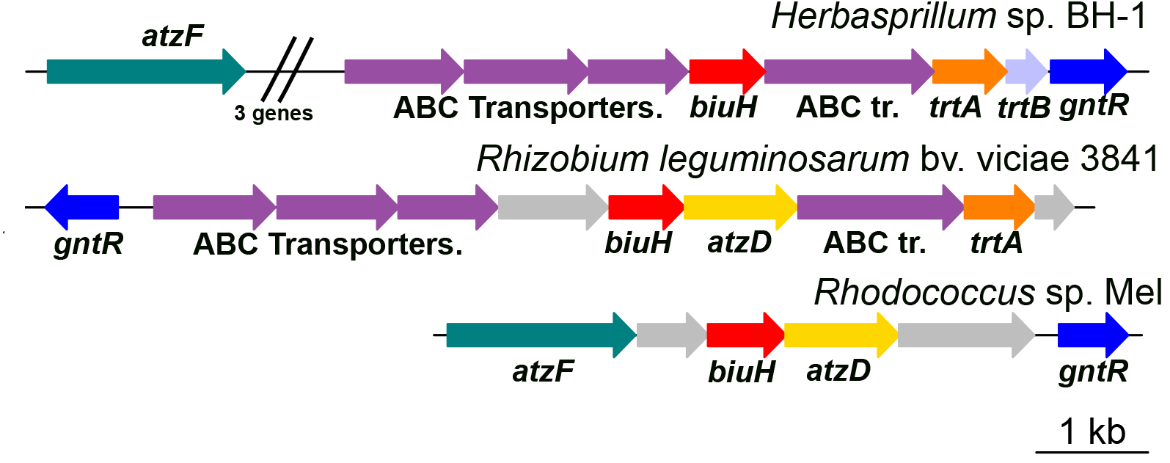
**

**Supp. Figure 2**

**
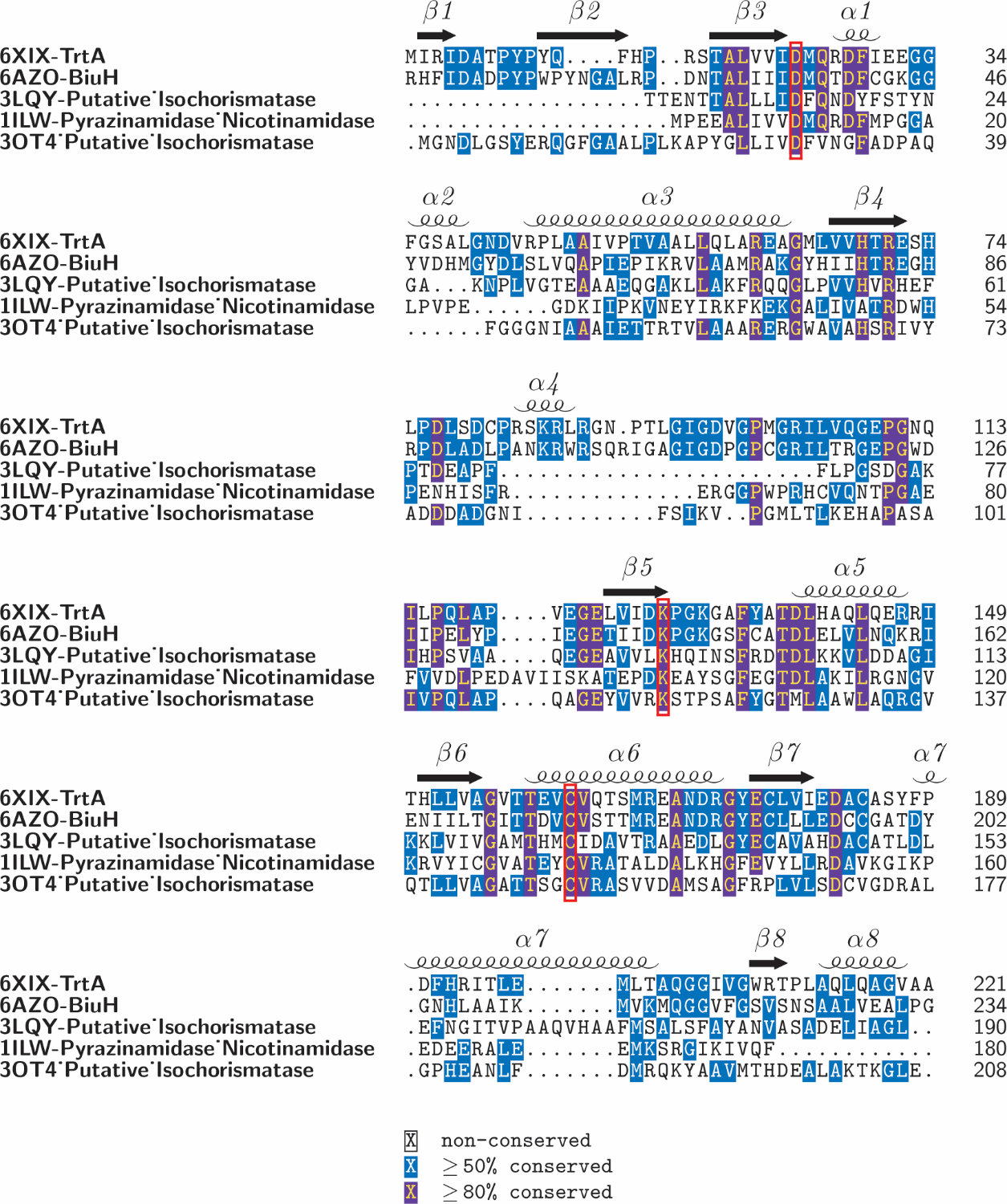
**

**Supp. Figure 3**


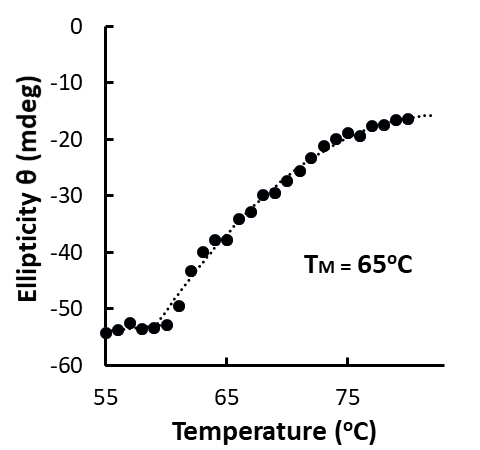


**Supp. Figure 4**

**
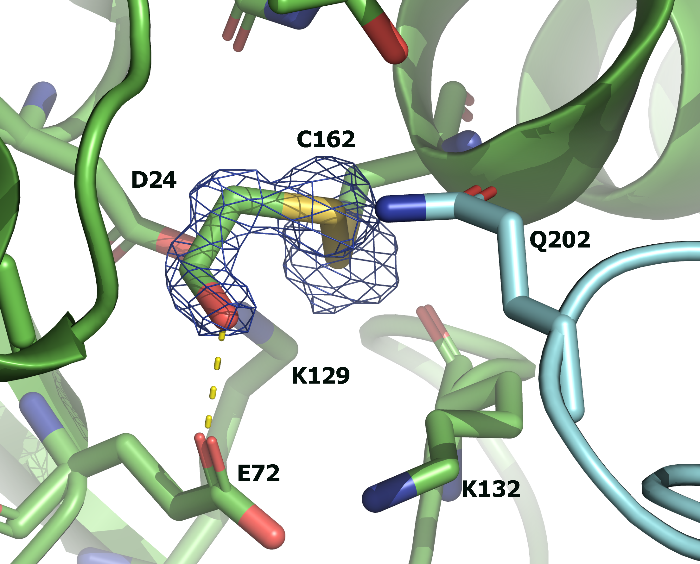
**

**Supp. Figure 5**

**
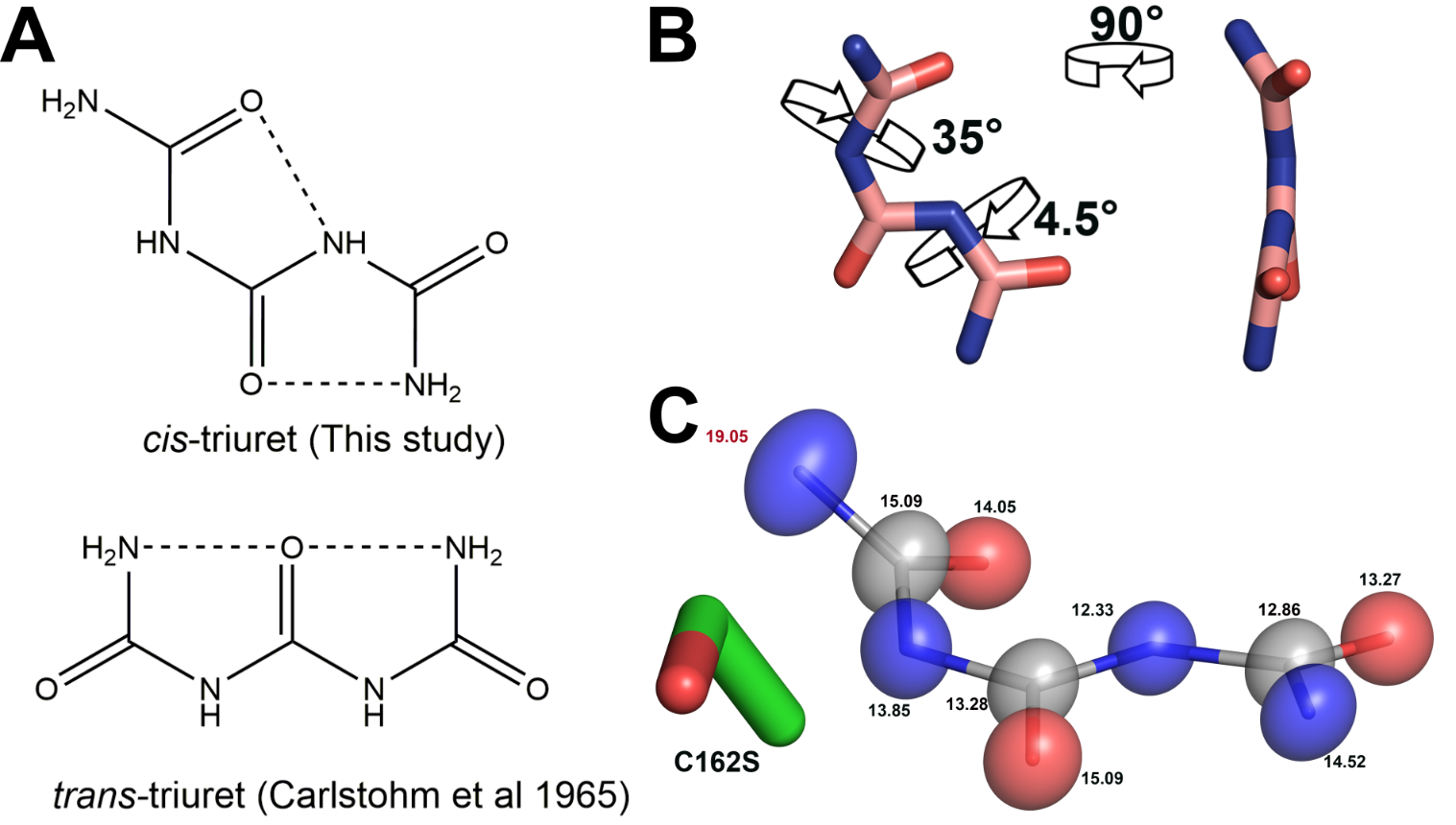
**

**Supp. Figure 6**

**
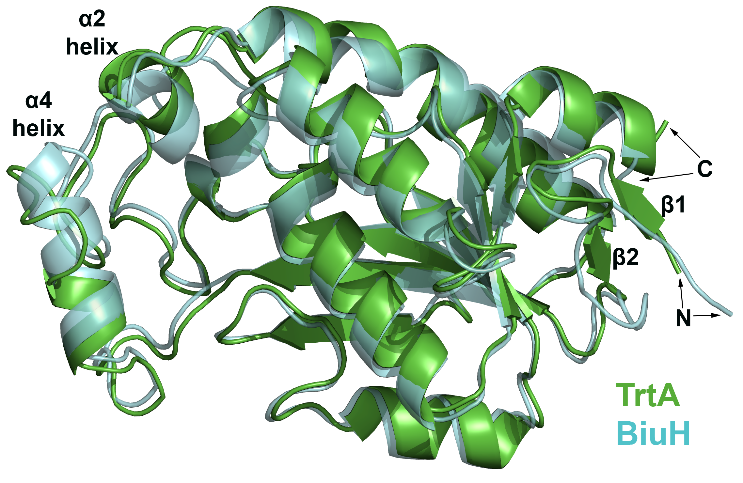
**

**Supp. Figure 7**

**
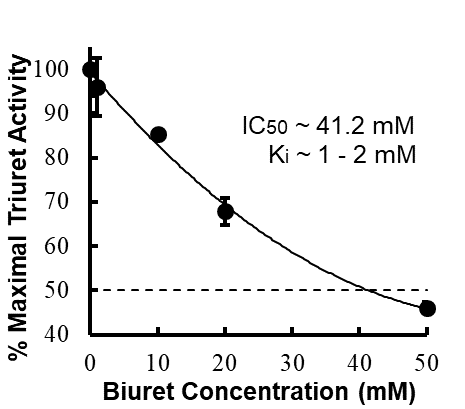
**

**Supp. Figure 8**

**
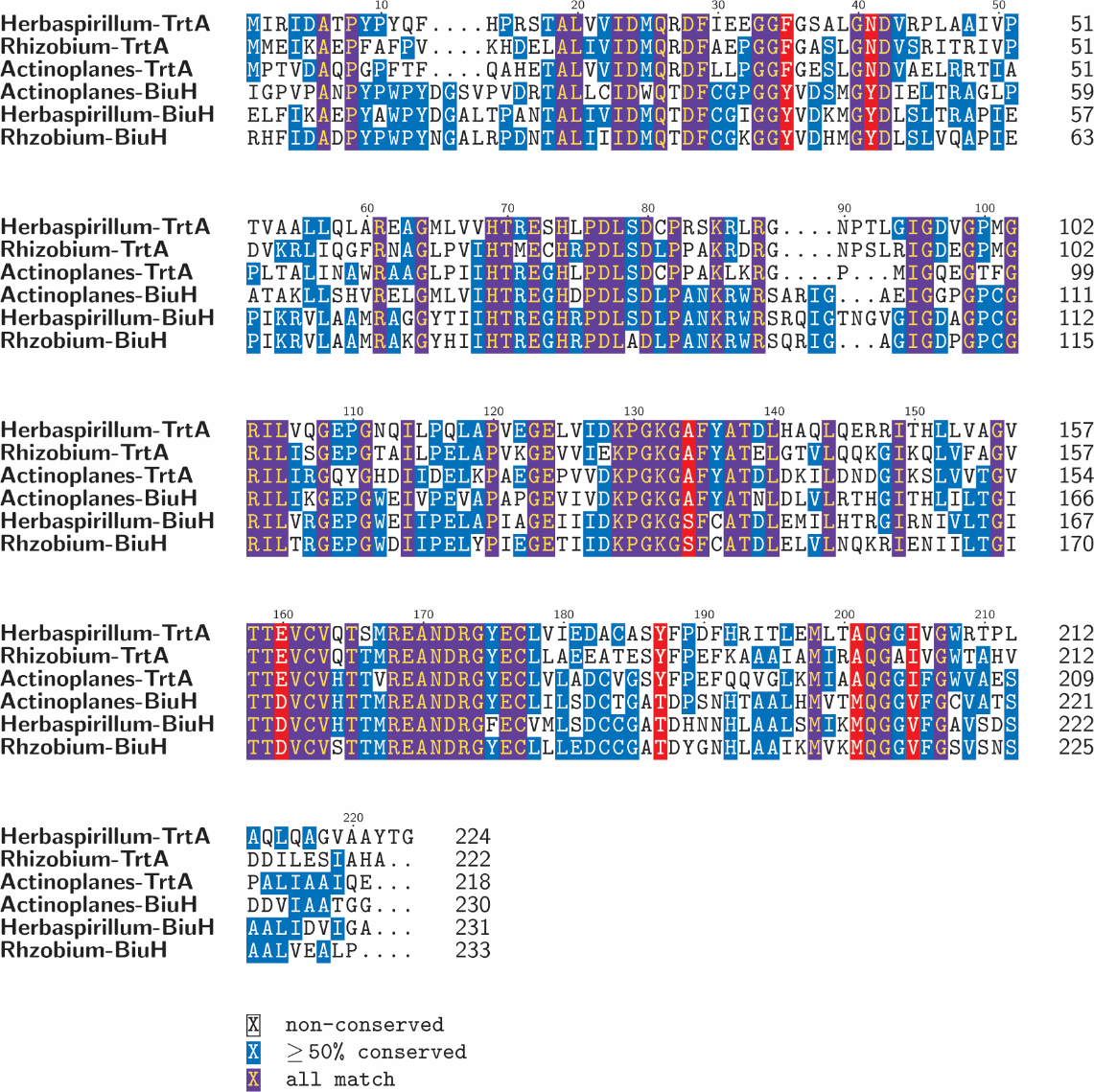
**

**Supp. Figure 9**

**
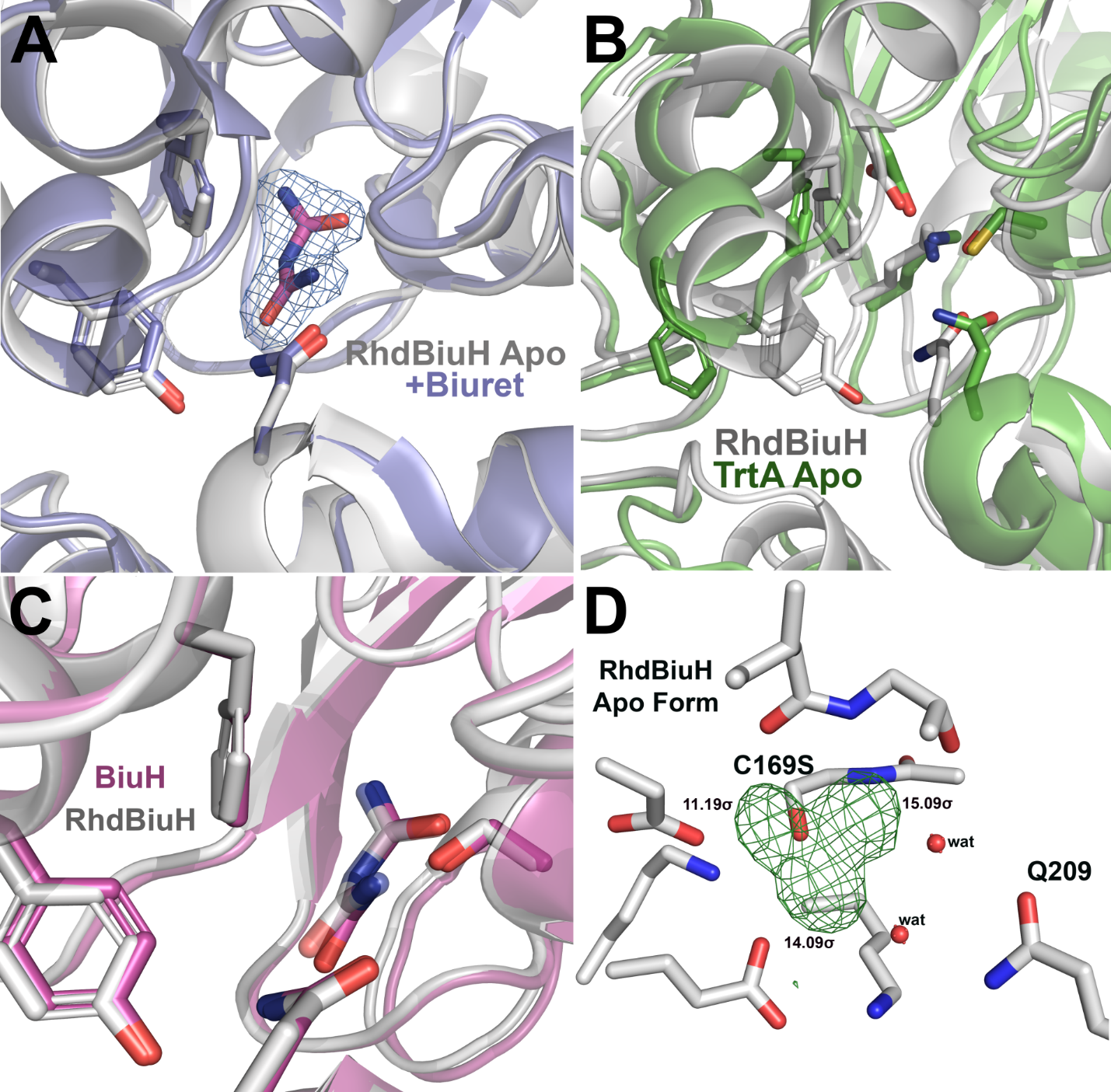
**
